# The complexity of inequity: rethinking global health through lived experience, power, and structural vulnerability

**DOI:** 10.1186/s12939-026-02810-5

**Published:** 2026-03-26

**Authors:** Yusuf Hared Abdi, Yakub Burhan Abdullahi, Naima Ibrahim Ahmed, Mohamed Sharif Abdi, Sharmake Gaiye Bashir, Ahmed Abdiaziz Alasow

**Affiliations:** Faculty of Medicine and Health Science, Hormuud University, Mogadishu, Somalia

**Keywords:** Health equity, Structural vulnerability, Complexity theory, Lived experience, Global health governance, Social determinants

## Abstract

Global health’s persistent inequities reflect not just gaps in resources or care, but the operation of complex, multi-level systems that inscribe power, vulnerability, and embodied experience. Prevailing frameworks remain too narrow, neglecting how health emerges from intersecting histories, adaptive institutions, and the lived experience of marginalized populations. This perspective advances a complexity-informed rethinking of inequity, integrating insights from structural vulnerability, epistemic justice, intersectionality, and the capability approach to illuminate how reductionist solutions overlook system dynamics and relational harms. By bridging data and narrative, and grounding analysis in both structural critique and lived experience, this work reframes inequity as emergent and fundamentally political. Case examples from maternal health and HIV stigma illustrate how structural arrangements and social meanings contour health in context-dependent ways. The implication is clear: achieving equity requires methodological humility, ethical rigor, and governance reforms that redistribute not only resources, but also epistemic authority and institutional power. This perspective offers an integrated conceptual architecture and calls for actionable transformation reorienting global health toward solidarity, partnership, and justice.

## Introduction

This perspective article examines global health inequity through a complexity-informed lens. Health inequity is not a simple arithmetic of missing resources or a tally of preventable deaths. It is a deeply layered, lived experience shaped by interlocking histories, shifting power arrangements, and the lived experience of people navigating circumstances they did not choose [1]. Behind every epidemiological statistic lies a human story a mother who walked hours for prenatal care that never came [2], a person living with HIV who encountered stigma instead of solidarity [3], a displaced family whose health needs became invisible in the architecture of emergency response [4]. Numbers matter: they reveal patterns, demand accountability, and marshal political will. Yet numbers alone cannot capture what it means to live within structures that distribute health and suffering unequally [5].

This paper examines global health inequity through a complexity-informed lens that honors both data and narrative, both systems and subjectivity. It argues that addressing inequity requires moving beyond reductionist models that locate health problems solely in individual behavior or isolated interventions, toward frameworks that recognize health as emergent from dynamic, multi-scalar systems [6]. Complexity theory, intersectionality, structural vulnerability, the capability approach, and critical analyses of power provide conceptual tools for understanding how inequity operates across multiple levels simultaneously [7].

These frameworks illuminate why well-intentioned interventions often fail, why small changes can produce large effects, and why context shapes outcomes in ways that linear models cannot predict [8]. By integrating lived experience with structural analysis, quantitative evidence with qualitative understanding, this paper proposes a more ethically grounded and practically effective approach to global health research, policy, and practice [9].

## Theoretical foundations

Understanding health inequity demands conceptual frameworks capable of grappling with simultaneity, interdependence, and emergence. Complexity science offers such a lens, viewing health outcomes not as products of isolated causes but as emergent properties of systems characterized by feedback loops, non-linear dynamics, and adaptive behavior [6]. In complex health systems, big effects can arise from seemingly small changes such as enabling women to control household resources in patriarchal settings while large-scale restructuring may yield disappointingly modest results [8]. This unpredictability underscores the limitations of linear cause-effect models and highlights the necessity of context-sensitive analysis that attends to patterns, relationships, and system dynamics rather than isolated variables [10].

Intersectionality, developed by Kimberlé Crenshaw and expanded within health research, illuminates how multiple axes of identity and structural position race, gender, class, disability, migration status shape health outcomes through overlapping systems of oppression [5]. Health inequities are rarely attributable to single factors; they arise from the compounding and interactive effects of social locations that distribute risk, resources, and recognition unequally [11]. Structural vulnerability extends this analysis by directing attention to the political, economic, and institutional arrangements that place populations at risk independent of individual behavior or cultural practices. Paul Farmer’s concept of structural violence captures how social structures themselves—such as poverty, racism, gender inequality—inflict harm, constrain agency, and produce premature death [12].

The capability approach, pioneered by Amartya Sen, shifts the evaluative focus from resources alone to what people are able to be and do with those resources [9]. Health is understood not merely as the absence of disease but as a fundamental capability that enables human flourishing [13]. This framework asks not only whether people have access to healthcare, but whether they possess the real freedoms to achieve health freedoms shaped by education, economic security, social participation, and political voice. Power—understood here as the ability to make decisions, control resources, and determine whose knowledge counts—operates across interpersonal, institutional, and structural levels. In health contexts, power asymmetries determine who has voice in policy, whose research questions are prioritized, and whose experiences are centered in knowledge production. Finally, analyzing power who decides, who benefits, whose knowledge counts is essential for understanding how inequities are produced and sustained [5]. Epistemic injustice operates through multiple mechanisms. Testimonial injustice occurs when a person’s testimony or knowledge claims are discredited based on prejudice or stereotype (for example, when healthcare workers dismiss HIV patients’ accounts of their treatment experiences based on stigma). Hermeneutic injustice emerges when communities lack adequate interpretive frameworks to communicate their experiences (for example, when mentally displaced populations cannot articulate trauma symptoms because available medical language does not recognize displacement-related psychological distress). Both forms reinforce patterns of marginalization and exclude marginalized populations from knowledge production [14–16]. These theoretical foundations together provide a robust conceptual architecture for examining health inequity as a multi-dimensional, structurally embedded, and dynamically evolving phenomenon.

## Lived experience

Inequity is not an abstraction; it is lived experience—embodied, felt, and narrated by those navigating its constraints. Nancy Krieger’s concept of embodiment describes how social conditions literally ‘get under the skin,’ manifesting as the lived experience of differential disease patterns, life expectancies, and biological expressions of disadvantage. Nancy Krieger’s concept of embodiment describes how social conditions literally “get under the skin,” manifesting as differential disease patterns, life expectancies, and biological expressions of disadvantage [1]. Bodies tell stories of the environments, exposures, stresses, and opportunities that shape lives from conception onward. For internally displaced persons, health vulnerabilities compound as displacement disrupts social networks, income sources, and access to care, while exposing populations to violence, food insecurity, and inadequate shelter [4, 17]. Studies reveal that displaced women face heightened risks of maternal mortality and morbidity, yet their health needs remain underserved by systems not designed for mobile, marginalized populations [18, 19].

Maternal health inequities similarly reflect structural failures rather than individual deficiencies. Women in low-resource settings navigate institutional delivery barriers including distance, cost, disrespectful care, and fear of surgical intervention [2]. Those who experience their first pregnancy before age twenty or lack antenatal care face disproportionate risks. Yet framing these as individual behavioral deficiencies obscures the structural conditions—poverty, gender inequality, and weak health systems—that constrain women’s choices and access to care. Women’s health outcomes reflect not personal failings but the cumulative effect of structural barriers they must navigate. HIV-related stigma operates as a structural barrier that transcends individual attitudes. Stigma is institutionalized through healthcare policies (such as patient isolation protocols), legal frameworks (criminalization of transmission), and religious/cultural discourses that have been weaponized to marginalize key populations. These structural dimensions ensure that stigma persists even as individual attitudes gradually improve, requiring systemic reforms in healthcare protocols, legislation, and public education rather than reliance on attitude-change campaigns alone. Such structural stigma shapes healthcare interactions, policy environments, and social relationships in ways that impede testing, treatment adherence, and viral suppression [3]. HIV stigma is not simply a problem of individual attitudes but is institutionalized through healthcare policies (such as patient isolation), legal frameworks (criminalization of transmission), and religious/cultural discourses that have been weaponized to marginalize key populations [3]. These structural dimensions ensure that stigma persists even as individual attitudes improve, requiring systemic reforms in law, healthcare protocols, and public education. Stigma enacted within healthcare settings correlates with suboptimal treatment adherence, demonstrating how interpersonal discrimination reflects and reinforces systemic marginalization [20].

## Systems and power structures

Health inequities are produced and sustained through systems operating at multiple scales, from global political economy to local governance structures. The political economy of health examines how economic policies, trade agreements, labor markets, and wealth distribution shape population health patterns and health system capacities [5]. Global health financing remains fragmented and donor-driven, with external funding often directed toward vertical disease-specific programs rather than integrated, country-owned health system strengthening [21]. While vertical programs have achieved notable successes in addressing HIV, tuberculosis, and malaria, their proliferation can undermine horizontal integration, create parallel reporting systems, and distort national health priorities according to donor preferences rather than local epidemiological profiles [22, 23].

Aid dependency introduces governance challenges: recipient countries may lack authority over programs operating within their borders, while conditionalities and short funding cycles constrain planning horizons and sustainability [21]. The tension between country ownership a principle enshrined in development effectiveness frameworks and donor power reflects deeper asymmetries in global health governance [24]. Democratic governance and accountability mechanisms remain underdeveloped in global health institutions, where decision-making authority concentrates among high-income country governments and philanthropic actors [25]. Calls for renewed global health governance emphasize principles of equity, transparency, inclusive participation, and solidarity, yet implementation lags behind rhetoric [26].

The digital divide further exacerbates inequity in the contemporary moment. While digital health technologies promise transformative potential, their deployment in low- and middle-income countries raises concerns about data colonialism, sovereignty, and epistemic justice [27]. Data colonialism—the extraction and commodification of data from populations in the Global South by external actors without meaningful participation or benefit-sharing—represents a contemporary form of resource extraction. African nations increasingly confront challenges related to data governance, algorithmic bias, and foreign control over digital infrastructure and data flows [28]. When health data generated from African populations is extracted, analyzed, and monetized by external actors without reciprocal benefit or meaningful participation in knowledge production, patterns of colonial extraction are reproduced in digital form [29]. Data inequality the unequal capacity to generate, access, analyze, and govern health data reinforces existing power asymmetries and limits the agency of populations and governments in the Global South [30]. Addressing these power dimensions of power requires systemic reforms that redistribute authority, resources, and epistemic standing within global health systems.

## Integrating data, narratives, and ethics

Moving beyond the false dichotomy between quantitative rigor and qualitative depth, contemporary methodological innovations emphasize integration as both epistemological necessity and ethical commitment [31]. Mixed methods approaches combine numerical data’s capacity to reveal patterns across populations with narrative’s power to illuminate meaning, context, and lived experience [32]. When applied thoughtfully, mixed methods can capture both the scale of inequity and its human texture, both systemic trends and individual agency [33]. Community-based participatory research grounds inquiry in partnership, co-designing research questions, methods, and interpretation with communities rather than imposing externally defined agendas [34]. This approach acknowledges that communities possess expertise about their own circumstances and that sustainable change emerges from collaborative processes that build local capacity and respect community priorities [35]. For instance, a study on maternal health might combine quantitative data on facility delivery rates by socioeconomic status with qualitative interviews exploring barriers women face, revealing both the scale of disparity and the lived constraints shaping women’s choices.

Realist evaluation, pioneered by Ray Pawson, offers a framework for understanding complex interventions by asking not merely “what works?” but “what works, for whom, in what circumstances, and why?” [36]. Rather than seeking universal best practices, For maternal health programs in resource-limited settings, realist approaches ask: What works (improved facility readiness)? For whom (women with transportation access)? In what circumstances (when midwives are trained and motivated)? This specificity prevents overgeneralizing findings across different contexts, realist approaches generate context-sensitive program theories that specify mechanisms the underlying processes through which interventions produce effects and how these mechanisms are activated or constrained by contextual features [37, 38]. This methodology is particularly valuable for evaluating health systems strengthening initiatives, where outcomes depend on interactions between intervention components, implementation contexts, and the responses of diverse stakeholders [39–41].

Systems thinking provides overarching principles for understanding health as produced by interdependent components whose interactions generate emergent properties [10]. Practical systems thinking tools include causal loop diagrams that map feedback mechanisms, systems archetypes that identify recurring patterns, and stakeholder engagement workshops that surface diverse perspectives on system dynamics. These tools make visible the interconnections often obscured by sectoral approaches to health policy [10]. Applying systems approaches to health equity requires mapping feedback loops, identifying leverage points, and anticipating unintended consequences [8]. Participatory action research methods directly address epistemic injustice by centering the knowledge and agency of marginalized populations, transforming them from research subjects into co-investigators [14]. Operationalizing these methodological commitments demands reflexivity about researcher positionality, power dynamics, and the colonial legacies embedded in knowledge production practices. Decolonizing global health research involves specific, measurable reforms: directly funding research institutions in low- and middle-income countries rather than channeling resources through high-income country intermediaries; elevating researchers from the Global South to decision-making and primary authorship positions (not token co-authorship); committing to sustained, long-term funding that supports institutional capacity building rather than short-term extractive partnerships; and ensuring that research is owned, controlled, and generates direct benefits to the communities studied [42, 43]. This decolonial approach explicitly redistributes control over research agendas, funding allocation, authorship, and knowledge dissemination, while cultivating research relationships grounded in reciprocity, respect, and solidarity.

Participatory action research in HIV prevention might involve people living with HIV co-designing research questions about barriers to treatment adherence, ensuring interventions address priorities identified by affected communities rather than predetermined by researchers.

## Case examples

The following examples illustrate how complexity-informed frameworks translate into practice. Each demonstrates the integration of structural analysis with relational care, moving beyond reductionist approaches to address the multi-level determinants of health inequity.

Accompaniment—a model of engaged, sustained partnership in healthcare—moves beyond service delivery to include social support, advocacy, and systemic change alongside clinical care. The accompaniment model developed by Partners In Health exemplifies complexity-informed practice that integrates structural intervention with relational care [44]. Implemented initially in rural Haiti and subsequently adapted in Rwanda, Peru, and other settings, accompaniment involves community health workers providing not only clinical services but also social support, advocacy, and connection to resources [45, 46]. In Rwanda, Partners In Health supported district-level health systems strengthening through mentorship, data-driven quality improvement, performance-based financing integrated with coaching, and peer-to-peer learning networks [47, 48]. These interventions recognized that improving health outcomes requires addressing multiple system levels simultaneously—such as facility infrastructure, supply chains, workforce capacity, community engagement, and governance [49]. Evaluations documented improvements in facility readiness, service delivery, and population health indicators, demonstrating the feasibility of comprehensive systems approaches even in resource-constrained settings. This approach exemplifies systems thinking by addressing multiple system levels and operationalizes structural competency by recognizing how facility infrastructure, supply chains, and governance structures shape health outcomes.

Social medicine initiatives in Latin America have long emphasized the structural determinants of health, linking clinical practice with advocacy for housing, nutrition, education, and political rights [12]. These approaches understand health as inseparable from social justice and position healthcare providers as allies in broader struggles for equity. Participatory action research addressing epistemic injustice in mental health offers another example, where researchers collaborated with individuals with lived experience to co-design studies, interpret findings, and disseminate results [14, 50]. This approach explicitly challenges testimonial and hermeneutic injustices where patients’ knowledge is dismissed or their experiences lack adequate interpretive frameworks by repositioning lived experience as foundational rather than supplementary to knowledge production [51].

## Implications for practice and policy

Translating complexity-informed analysis into actionable strategies requires multi-level interventions that address individual, community, health system, and policy domains simultaneously [52]. Public health systems must move beyond disease-focused vertical silos toward integrated, person-centered care that addresses social determinants alongside biomedical needs [22, 53]. This integration demands flexible governance structures, interoperable data systems, and financing mechanisms that reward coordination rather than competition among programs. Structurally competent clinicians recognizing a patient’s poor medication adherence might explore structural barriers—such as cost of transportation to pharmacy, work schedule conflicts, or discrimination encountered in healthcare settings—rather than attributing nonadherence to patient motivation or health literacy. Training healthcare professionals in structural competency equips them to recognize and respond to how political, economic, and institutional forces shape patient health and healthcare access [54, 55]. Structural competency curricula teach clinicians to identify upstream factors contributing to health disparities, understand their own positionality within power structures, and develop advocacy skills to address systemic barriers [11, 56, 57].

Narrative medicine, championed by Rita Charon, cultivates clinicians’ capacity for attentive listening, empathetic engagement, and recognition of illness as lived experience rather than biological malfunction alone [58, 59]. Integrating narrative approaches into medical education fosters humility, reflexivity, and patient-centered communication [60–62]. Communication strategies within public health must balance epidemiological data with storytelling that conveys complexity, honors dignity, and avoids stigmatizing representations. Global health governance reforms should prioritize democratic participation, accountability, transparency, and the principle of “nothing about us without us,” ensuring that affected populations meaningfully shape decisions that impact their health [24, 25].

Addressing digital health inequities requires investments in data sovereignty, capacity building for local data science expertise, and ethical governance frameworks that protect privacy while enabling innovation [28, 30]. Health research funding should incentivize partnerships that build sustained institutional capacity in low- and middle-income countries rather than extractive collaborations that concentrate benefits in high-income settings [43, 63]. Operationalizing these recommendations demands humility about the limits of external expertise, commitment to long-term solidarity over short-term deliverables, and willingness to redistribute power and resources in ways that challenge entrenched hierarchies [64, 65].

- This requires establishing mechanisms for meaningful participation of affected populations in policy decisions (moving beyond consultation to co-governance).
- Implementing transparent allocation of resources from global health institutions”.
- Redistributing research agenda-setting authority to researchers and institutions in low- and middle-income countries.

## Conclusion

Rethinking global health through the lenses of complexity, lived experience, power, and structural vulnerability reveals inequity not as a technical problem awaiting linear solutions, but as an emergent, multi-dimensional phenomenon demanding ethical engagement and systemic transformation. This paper has argued that meaningful progress requires integrating quantitative and qualitative knowledge, centering the voices of those experiencing inequity, analyzing structural determinants across scales, and cultivating methodologies grounded in partnership, reflexivity, and epistemic justice. The challenge is not merely intellectual but profoundly ethical: to confront the ways global health itself has perpetuated inequities through colonial legacies, extractive research practices, and power asymmetries embedded in governance and financing structures.​.

Understanding health inequity as a complex phenomenon does not paralyze action; rather, it enables more effective and humane interventions. Complex systems contain multiple points of leverage where seemingly small changes can cascade across system levels. By recognizing feedback loops, unintended consequences, and context-specificity, policymakers and practitioners can design interventions that work with system dynamics rather than against them, increasing the likelihood of sustainable change.

Moving forward requires humility about what we do not know, courage to question dominant paradigms, and commitment to solidarity that transcends charity. It demands recognition that health is political, that knowledge production is never neutral, and that achieving equity requires redistributing not only resources but also authority, voice, and epistemic standing. The complexity of inequity resists simple solutions, yet this complexity also reveals multiple points of intervention and transformation. By honoring both the irreducible dignity of lived experience and the structural forces shaping that experience, global health can evolve toward practices that are not only more effective but also more just, more humble, and more deeply human.

This transformation is not merely aspirational but urgently necessary. Funders must redirect resources toward equitable North-South partnerships. Policymakers must establish mechanisms for meaningful participation of affected communities. Researchers must practice epistemic humility, recognizing that those experiencing inequity possess essential knowledge for its transformation. The complexity of inequity demands nothing less than comprehensive, systemic, and fundamentally political change.

## Data Availability

No datasets were generated or analyzed for this manuscript. All materials are conceptual in nature and are derived from previously published peer-reviewed literature cited in the manuscript.
